# Nuclear DNA Content Analyses by Flow Cytometry of Saffron (*Crocus sativus* L.) Populations Obtained from Safranbolu, Türkiye

**DOI:** 10.3390/cimb48030262

**Published:** 2026-03-01

**Authors:** Gülru Yücel, Şahane Funda Arslanoğlu, Ogün Demir, Bozena Kolano, Metin Tuna

**Affiliations:** 1 Department of Agricultural Biotechnology, Faculty of Agriculture, Ondokuz Mayis University, 55139 Samsun, Türkiye; 2 Department of Field Crop, Faculty of Agriculture, Ondokuz Mayis University, 55200 Samsun, Türkiye; farslanoglu@omu.edu.tr; 3 Department of Biodiversity and Information, Nezahat Gökyiğit Botanic Garden, 34758 İstanbul, Türkiye; ogundemir8@gmail.com; 4 Plant Cytogenetics and Molecular Biology Group, Institute of Biology, Biotechnology and Environmental Protection, Faculty of Natural Sciences, University of Silesia in Katowice, 40-032 Katowice, Poland; bozena.kolano@us.edu.pl; 5 Department of Field Crops, Faculty of Agriculture, Tekirdağ Namık Kemal University, 59030 Tekirdağ, Türkiye; mtuna@nku.edu.tr

**Keywords:** *Crocus sativus*, Saffron, flow cytometry, nuclear DNA content

## Abstract

*C. sativus* (saffron) is the source of the world’s most expensive spice. Despite its economic significance, the genome structure is poorly studied. *C. sativus* is a sterile triploid (2*n* = 3*x* = 24) species, traditionally considered to exhibit minimal genetic variation. In this study, we analysed 45 individuals representing 15 accessions of *C. sativus* obtained from farmers in the Davutobası and Yukarıçiftlik villages of Safranbolu—an important centre of saffron cultivation in Türkiye. These populations represent an underexplored reservoir of germplasm with potential implications for biodiversity, conservation, and genetic improvement. Flow cytometry based on propidium iodide staining was used to assess nuclear DNA content, a key cytogenetic characteristic relevant to taxonomy, breeding, and molecular research. Nuclear DNA content among individuals ranged from 10.45 pg/2C DNA to 10.9 pg/2C DNA, all sharing the expected triploid chromosome number (2*n* = 3*x* = 24). Although variation was subtle, the observed polymorphism suggests the presence of detectable genomic diversity within these genotypes. These findings highlight the importance of analysing genotypes in understanding the genetic landscape of *C. sativus*. Selected individuals exhibiting variation in genome size may serve as valuable material for further molecular and breeding studies aimed at improving this culturally and economically significant crop.

## 1. Introduction

Saffron (*Crocus sativus* L., Iridaceae Juss.) is the source of the world’s most expensive spice [1,2,3,4], which is extracted from the dehydrated stigmas of the flower [3]. The genus *Crocus* includes more than 235 species, distributed from Poland to China, with most species growing in Türkiye and the Balkans [5]. *C. sativus* is a sterile herbaceous perennial plant propagated vegetatively by bulbs [1,6]. Saffron is primarily cultivated in Iran, Spain, India, Italy, and Greece [7]. In Türkiye, it is less widely grown, and its cultivated area is mainly restricted to Safranbolu [8,9]. It is stated that saffron’s homeland is Anatolia and the surrounding area of the Eastern Mediterranean region; however, according to other sources, saffron was brought to Anatolia from Central Asia. It was known that saffron was produced in 40 villages of the Safranbolu district [9,10,11]. Currently, saffron cultivation continues only in three villages, namely Davutoba, Aşağı Güney, and Yörük, covering an area of 4310 m^2^ [9]. Thus, it is believed that saffron gave rise to the name Safranbolu [10,11]. Due to advancements in dye technology and the pharmaceutical business’s high cost, the consumption has drastically decreased. However, owing to saffron’s significant economic value and considerable global demand, its cultivation remains an important issue [11].

The origin of *C. sativus* (allotriploid or autotriploid) has been debated for a long time. Recent cytogenetic studies have shown that saffron is an autotriploid species derived from *C. cartwrightianus* [1]. *C. sativus* has a chromosome number of 2*n* = 3*x* = 24 [1,12]. Due to irregularities during meiosis that lead to non-viable gametes and the absence of seeds, saffron is propagated only vegetatively via its bulbs [13,14]. It has been hypothesised that its clones are genetically similar [15]. Indeed, analyses based on molecular markers have revealed very limited genetic variation among *C. sativus* individuals, e.g., AFLP [15], iPBS [16], ISSR, and RAPD [17]. In addition, a recent study on the genetic diversity and population structure of *C. sativus* reported genetic variation based on SRAP analyses [18]. Consequently, traditional breeding approaches such as selection and crossbreeding cannot be effectively used to genetically improve *C. sativus* or develop new varieties with desired traits [14]. Therefore, assessing the level of genetic diversity within *C. sativus* is crucial for developing effective biodiversity conservation strategies and crop improvement programmes [15]. Previously reported that nuclear genome size may reinforce biodiversity evaluation and conservation studies, which is why it is considered important knowledge [19,20]. On the other hand, it has been documented that inducing chromosome doubling may offer opportunities for genetic enhancement through breeding. However, to date, such efforts have only produced hexaploid cytotypes under in vitro conditions [13]. Polymorphism in nuclear DNA content is one of the important and relatively easy-to-assess factors of intraspecific diversity [21,22]. Nuclear genome size is a fundamental biological characteristic of all living organisms and a key karyological trait required in various fields of biology, such as ploidy analyses, taxonomy, breeding studies, and genome analyses [23,24,25]. For a long time, the amount of DNA in nuclei was considered a constant characteristic among individuals of the same species and ploidy level [26]. However, the development of flow cytometry has enabled rapid and cost-effective estimation of DNA content in numerous individuals within a single species [27,28,29]. This advancement has allowed researchers to study genome size variation among numerous samples from different populations of a single species, revealing that significant genome size polymorphism exists in many species [30,31,32]. Such variation can serve as an indicator of intraspecific diversification among populations (*Chenopodium quinoa*; [32]), cultivars (*Camellia sinensis* var. *assamica*; [33]), landraces (maize; [34]) or varieties (apple; [29]). Examples of DNA content polymorphism have been observed in sunflower [30], maize [35], and *Chenopodium quinoa* [32]. Intraspecific variation in DNA content can be associated with variation in chromosome number, such as B chromosomes, polyploidy, and variation in the content of repetitive sequences [36,37,38,39,40]. A few articles reported estimates of nuclear DNA content in saffron [12,41,42]. However, different results were obtained in various studies, e.g., 9.75 pg/2C, 9.77 pg/2C DNA [41,42] and 11.35 pg/2C DNA [12]. Since these genome size estimates have been obtained from different experiments using various methods, standards, etc., it remains unclear whether the observed differences are due to technical issues or reflect true nuclear DNA content polymorphisms among saffron populations or individuals. Therefore, this study aims to determine whether *C. sativus* genotypes from Safranbolu exhibit polymorphisms in nuclear DNA content and whether this trait can be a marker for describing genetic variation within the species. The findings will contribute to the conservation and preservation of saffron’s genetic diversity, a genotype well adapted to its cultivation region. Additionally, the chromosome number of the analysed individuals was examined to rule out chromosomal variation as a potential source of differences in DNA content.

## 2. Materials and Methods

### 2.1. Material

**Figure 1:**
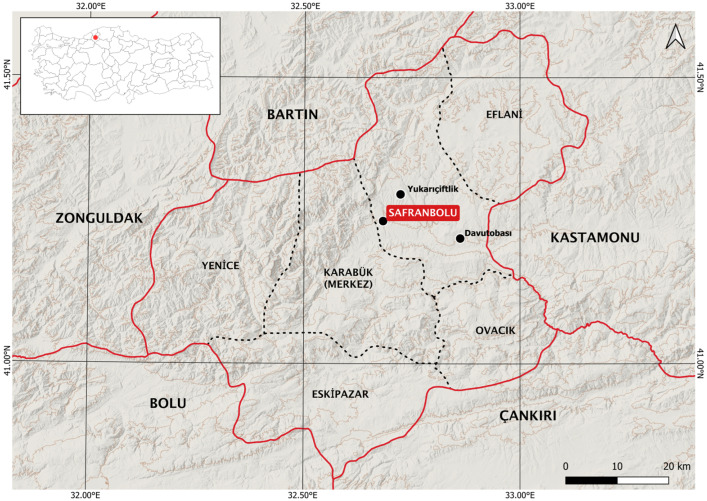
The locations where bulbs were obtained in Safranbolu are shown on the map.

Healthy, fresh bulbs of saffron genotypes grown in the region for a long time were obtained from 15 farmers in the Safranbolu Davutobası and Yukarıçiftlik villages. Safranbolu is a district located in the Western Black Sea region of northern Türkiye [11] and the main source centre of saffron output in Türkiye (Figure 1). The map was created using QGIS 3.34.9, an open–source geographic information system. The bulbs were harvested at the beginning of the summer and stored in cloth bags in a cold storage facility with a ventilation system at +4 °C and 60% humidity till use.

In August, the bulbs were removed from storage and kept in 80–85% moistened germination containers at 24 °C for 5 days to break dormancy. Following this treatment, 10 bulbs were taken from each supplier (a total of 150 bulbs) and planted in 10-litre horizontal pots filled with peat. Plants were grown from bulbs in a greenhouse facility of the Agricultural Faculty at Ondokuz Mayıs University under a 12 h/12 h photo-period at 18–24 °C with irrigation.

### 2.2. Sample Preparation for Flow Cytometry Analyses

For flow cytometry analysis, healthy, young, and fresh leaf tissues were used. Fresh leaf samples from each genotype were collected from three bulbs per genotype. Nuclear DNA content analysis of the samples was performed by flow cytometry (Partec CyFlow^®^ Space, Munster, Germany) in the Plant Genetics and Cytogenetics Laboratory at Tekirdağ Namik Kemal University. The CyStain PI Absolute P commercial kit, manufactured by Sysmex Partec GmbH (Münster, Germany), was used to prepare samples for flow cytometry analysis. *Vicia sativa* (3.65 pg/2C DNA; [29]) was used as an internal standard in the analyses. The youngest fully developed leaf tissues from both the sample and standard plant were placed in a Petri dish containing 500 µL of nuclei isolation buffer and chopped together using a razor blade into small pieces. The suspension was transferred through a 30 µm mesh in a tube and stained with 2000 µL of the dye solution (CyStain PI Absolute P). The samples were run in a flow cytometer following a 60 min incubation in the dark. The flow cytometry results were processed with the FloMax analysis software program [Version 2.81, Sysmex Partec GmbH (Münster, Germany)]. Nuclear DNA content of the analysed samples was calculated from the ratio of the G1 peak means of each sample and the internal standard using the following formula [27].

$$\begin{array}{c} \mathrm{Nuclear}\;\mathrm{DNA}\;\mathrm{content}\;\mathrm{of}\;\mathrm{the}\;\mathrm{Sample}\\ \;=\frac{Fluorescence\;intensity\;of\;sample\;(mean\;of\;G1\;peak)}{Fluorescence\;intensity\;of\;standart\;(mean\;of\;G1\;peak)}\\ \;\times \;DNA\;content\;of\;standard \end{array}$$

### 2.3. Somatic Chromosome Preparation

Approximately 2 cm long root tips were collected from growing bulbs, immersed in ice-cold water at 4 °C for 25 h, and fixed in a mixture of ethanol/glacial acetic acid (3:1). The somatic chromosome preparations of the sample root meristems were made as described by Hasterok et al. [43]. Shortly, fixed roots were washed in 0.01 M citrate buffer and then digested in a mixture of enzymes: 20% (*v*/*v*) pectinase (Sigma, Sigma, St. Louis, MO, USA) and 1% (*w*/*v*) cellulase (Sigma St. Louis, MO, USA) for 2 h at 37 °C. Dissected root tips of the samples were transferred to a microscope slide, placed in a drop of 45% acetic acid, and then squashed. The slides were examined in contrast phase microscopy, and images were captured with a digital camera (Toupcam E31SPM20000KPA, Touptek, Hangzhou, Zhejiang, China) attached to an Olympus BX51 microscope (Olympus Corporation, Tokyo, Japan).

### 2.4. Statistical Analysis

The statistical analyses were conducted using Python (version 3.11.9). Parametric tests were performed with the scipy (version 1.13.1) and statsmodels (version 0.14.4) libraries [44,45], while data manipulation and visualisation were carried out using pandas (version 2.1.4), matplotlib (version 3.9.1), and seaborn [46,47]. The significance level for all statistical procedures was set at α = 0.05. Prior to applying parametric tests, the distributional characteristics of nuclear DNA content data for each population were evaluated. Normality was assessed using the Shapiro–Wilk test [48], and the homogeneity of variances was tested using Levene’s test [49]. As the assumptions of normality and homoscedasticity were met, a one-way analysis of variance (ANOVA) was applied to examine whether mean nuclear DNA content differed significantly among populations [50,51,52]. When the ANOVA was significant, post hoc pairwise comparisons were conducted using Tukey’s Honestly Significant Difference (HSD) test to identify the specific population pairs contributing to the observed differences [52,53]. To assess similarity relationships among the 15 populations, a hierarchical cluster analysis was performed [54,55]. Pairwise dissimilarities between populations *A* and *B* were quantified using the Euclidean distance, which, for a single quantitative variable, reduces to

$$d_{AB}=|\mu_{A}-\mu_{B}|,$$

where *μ_A* and *μ_B* denote the mean nuclear DNA contents of populations A and B, respectively.

Clustering was carried out using the Unweighted Pair Group Method with Arithmetic Mean (UPGMA). When clusters i and j were merged to form a new cluster u, the distance between u and any other cluster k  was updated using the standard arithmetic-mean linkage function:

$$d_{uk}=\frac{N_{i}d_{ik}+N_{j}d_{jk}}{N_{i}+N_{j}},$$

where $N_{i}$ and $N_{j}$ represent the number of elements in clusters i and j, respectively. The resulting dendrogram provides a hierarchical representation of similarity patterns among the populations based on variation in their nuclear DNA content.

## 3. Results

**Figure 2:**
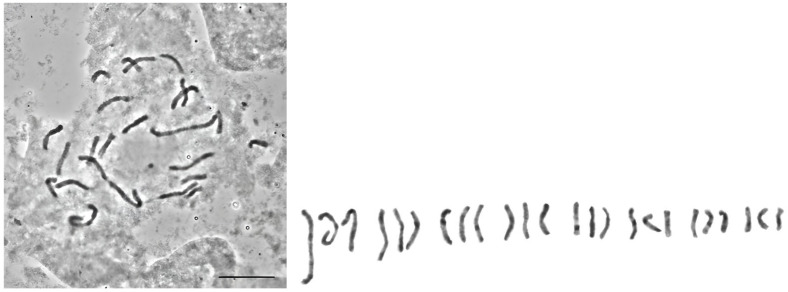
Somatic chromosomes of *C. sativus*.

The nuclear DNA content was estimated for 15 populations from the Safranbolu region in Türkiye. All analysed individuals were triploids with 2*n* = 3*x* = 24 (Figure 2). The somatic chromosomes were cut and shown alongside the metaphase plates. The *C. sativus* karyotype comprises various chromosome types: triplets 1 and 2 are subtelocentric, triplets 3, 4, and 8 are metacentric, while triplets 6 and 7 contain submetacentric chromosomes. Triplet 5 includes one metacentric chromosome and two subtelocentric chromosomes [1,56]. Between the longest and shortest chromosomes, there is a significant difference, which agrees with previous reports [56].

**Figure 3:**
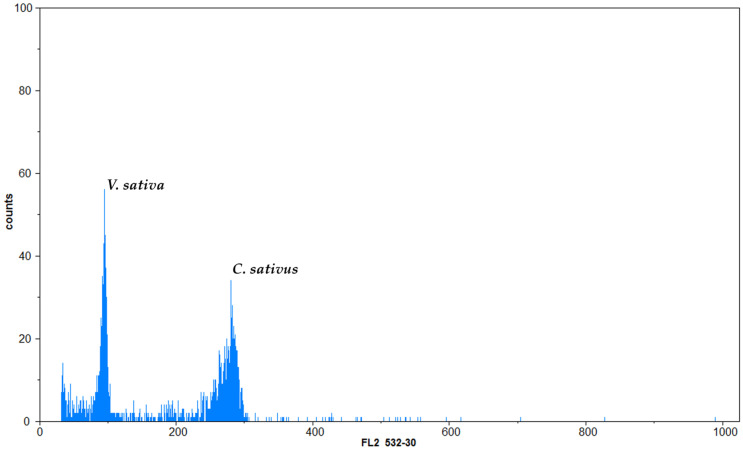
The flow cytometry histogram shows the relative positions of the G1 peaks of *C. sativus* and the standard. In the histogram, peaks corresponding to the G1 phases of *C. sativus* and *V. sativa* were shown, respectively. The flow cytometry results were processed using the FloMax analysis software (Sysmex Partec GmbH, Münster, Germany), which offered mean fluorescence intensity values. The nuclear DNA content of the analysed samples was calculated based on the ratio of the G1 peak means of each sample to that of the internal standard, using the formula proposed by [27].

Flow cytometric analysis generated good-quality histograms for each individual (Figure 2). The histograms of nuclear DNA content in young leaves showed a single distinct peak corresponding to G1 (2C DNA content) nuclei. *V. sativa* L. was a proper internal standard since its G1 peak was clearly distinguishable from the G1 peak of saffron (Figure 3).

The standard deviation of DNA content in the populations ranged from 0.07% to 0.38%. The mean 2C nuclear DNA content ranged from 10.45 pg/2C DNA to 10.9 pg/2C DNA. This corresponds to a 4,13% difference in DNA content between the analysed populations, with population no. 6 (from Davutobası) having the smallest DNA content and the 11th population (Davutobası) having the largest DNA content. An overview of the nuclear DNA content of the analysed genotypes is presented in Table 1. The mean 2C DNA content of the analysed saffron genotypes was 10.72 ± 0.14 pg.

**Figure 4:**
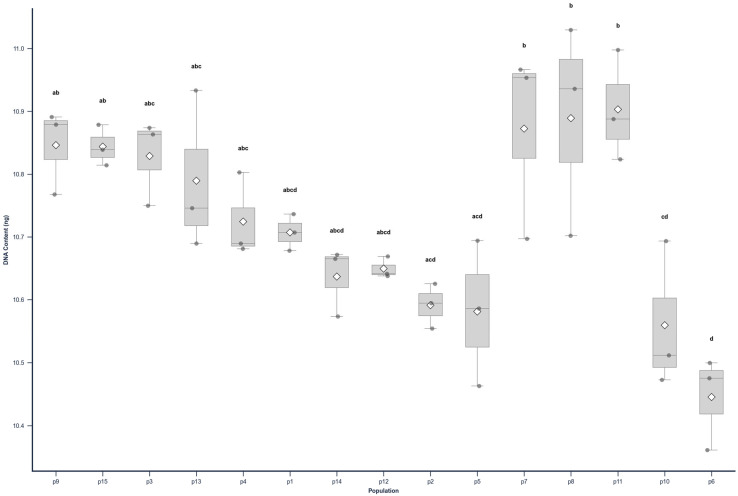
Boxplots illustrating the variation in 2C nuclear DNA content (pg) across 15 *C. sativus* populations. The grey line within each box represents the median, the diamond (◇) represents the mean, and the dots represent individual measurements. The letters (a–d) above the boxes denote statistically significant differences according to Tukey’s HSD post hoc test (*p* < 0.05); populations sharing the same letter do not differ significantly.

The distribution of nuclear DNA content (2C value) among the 15 analysed *C. sativus* populations is presented in Figure 4. The assumptions for parametric analysis were evaluated prior to hypothesis testing; the Shapiro–Wilk test indicated no departure from normality (*p* = 0.5586), and Levene’s test confirmed homogeneity of variances among populations (*p* = 0.9275). Consequently, a one-way ANOVA revealed statistically significant differences in mean nuclear DNA content across the dataset (F = 7.0541, *p* < 0.0001).

Post hoc comparisons using Tukey’s HSD test are visualised in Figure 4 using a compact letter display. Different letters above the boxplots indicate statistically significant differences (*p* < 0.05). The analysis revealed a clear separation between genotypes: population 6 (labelled ‘d) had the lowest mean nuclear DNA content (10.45 pg), which differed significantly from the higher DNA content group (populations 7, 8, and 11, labelled ‘b’). Population 10 also clustered with the lower range genotypes (‘cd’), showing significant divergence from the highest values. Populations sharing the same letter (e.g., p1, p12, p14) did not differ statistically, indicating a gradient of intermediate genome sizes rather than discrete, isolated groups for the majority of the samples.

**Figure 5:**
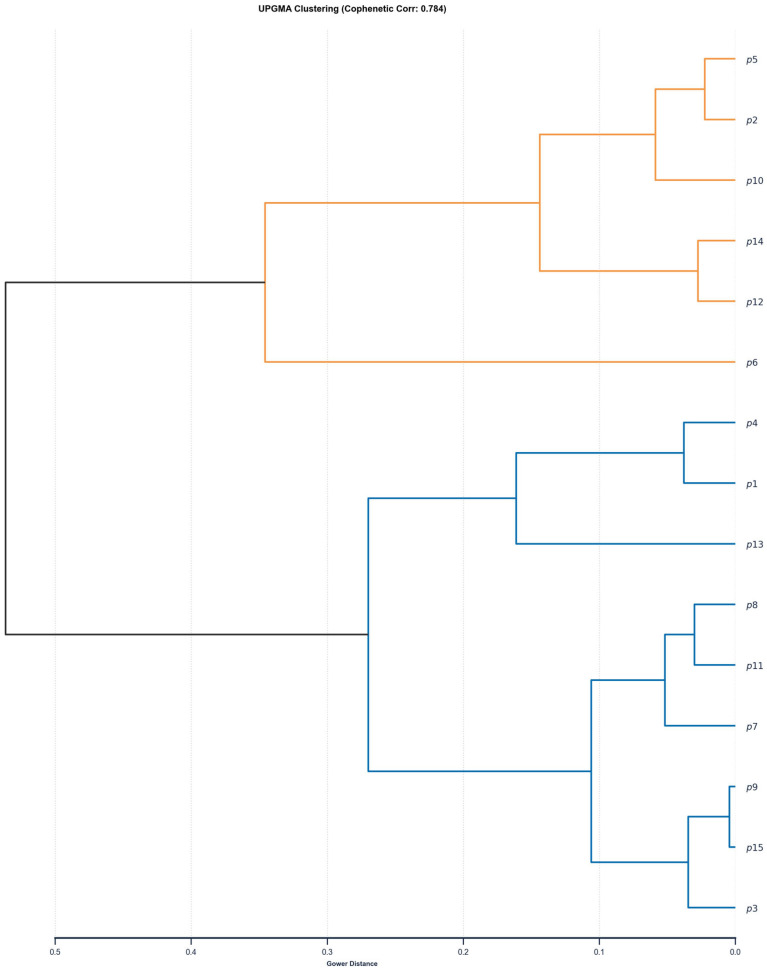
UPGMA dendrogram illustrating the clustering of 15 *C. sativus* populations based on Euclidean distances calculated from nuclear DNA content. The analysis reveals two major clusters: a “Higher DNA” clade (blue) and a “Lower DNA” clade (orange). The cophenetic correlation coefficient is 0.784.

To further explore the similarity structure among populations, hierarchical cluster analysis was performed using Euclidean distances and the UPGMA algorithm (Figure 5). The resulting dendrogram (cophenetic correlation = 0.784) resolved the populations into two main clades. The first clade (blue branches) predominantly indicated populations with relatively high nuclear DNA content, including populations 7, 8, 11, and 15. The second clade (orange branches) cluster populations characterised by lower mean values, such as populations 2, 5, 10, and 12. Notably, population 6 forms a basal, isolated branch within the second clade, reflecting its uniquely low nuclear DNA content and consistent statistical separation from other populations, effectively mirroring the ANOVA results.

Together, the ANOVA and clustering analyses indicate biologically meaningful differentiation in nuclear DNA content among the studied *C. sativus* populations.

## 4. Discussion

Nuclear genome size is a species-specific characteristic, and its precise estimation has been commonly performed in various species using flow cytometry with nuclei stained with propidium iodide [29,57]. From a different perspective, flow cytometry analyses based on PI staining are performed to compare nuclear DNA content variation among different genotypes within a single species, or even across related species [29,32]. This is the first study reporting 2C nuclear DNA content analyses of saffron growing in Safranbolu to detect possible variation among the 15 genotypes using propidium iodide by flow cytometry. Previously, a few reports were conducted to determine nuclear DNA content in *C. sativus*, which reported differences, e.g., 11.35 pg/2C [12], 9.75 pg/2C DNA [41], 9.77 pg/2C DNA [42]. In the present research, nuclear DNA content ranged from 10.45 pg/2C to 10.49 pg/2C). In contrast, previously obtained data showed slightly lower and higher variation in nuclear DNA content, ranging from approximately 9.75 pg/2C (samples from Türkiye and India) to 11.35 pg/2C (samples from Italy, Israel, and Spain) in saffron populations [12,41,42]. The differences in genome size reported across studies may be influenced by variations in standards, staining methods, genotype, and other methodological factors [58,59,60]. Devi et al. [42] reported 9.77 pg/2C DNA using *Secale cereale* and propidium iodide, while Erol et al. [41] found 9.75 pg/2C DNA using *V. sativa* analysed saffron from India and Türkiye, respectively. In contrast, Brandizzi and Caiola [12] reported a higher value of 11.35 pg/2C using human leukocytes and ethidium bromide (saffron from Italy, Israel, and Spain). While these differences may result from methodological variation, they may also reflect true polymorphisms in genome size. In fact, our results showed that polymorphisms in nuclear DNA content among individuals from saffron populations were statistically significant and reached up to 19.4% differences, supporting the idea that some of the observed variation may represent genomic diversity rather than technical artefacts. Since all analysed populations in this study have been assumed to be 2*n* = 3*x* = 24 chromosome number, observed differences in genome size might be caused by different amounts of repetitive sequences, mostly retrotransposons, which are a well-known source of genome size differences [34,61,62,63]. Intraspecific polymorphism in genome size content among individuals of the same species and ploidy level has been described in several species.

Notably, high variation in genome size has been reported in *Tulipa montana*. Despite having the same chromosome number (2*n* = 24), the genome size in this species ranged from 33.03 pg/2C to 51.36 pg/2C, depending on the individual [64]. Similar results were found in some genotypes of *Prospero autumnale*, where nuclear DNA content in diploid individuals ranged from 4.23 pg/2C DNA to 7.85 pg/2C DNA [65]. However, other species, such as *Chenopodium quinoa* (5.9%; [32]) and *Sesleria albicans* (1.6%; [66]), have shown much lower intraspecific genome size polymorphism, similar to the range observed in the present study for saffron. Genome size variation has also been observed in *Zea mays* L., likely related to the contribution of non-coding repetitive DNA sequences [34]. Also, a study on *Brassica rapa*, based on comparative genomics and cytogenetic approaches, showed the potential impact of differential insertion of repetitive elements on intraspecific genome size variation [63]. Several studies have reported that intraspecific genome size variation may result from different mechanisms, such as polyploidy [29] or changes in LTR retrotransposon content within the genome [61]. It has been reported that the genome size differences in the analysed plants are accompanied by variations in LTR retrotransposon content, implying that LTR retrotransposons can be essential players in plant genome size evolution. A burst of retrotransposons may increase in genome size, whereas counteracting mechanisms may eliminate retrotransposon copies caused by illegitimate recombination and unequal homologous recombination [61,67,68]. In our manuscript, we referenced various species in which genome size variation has been shown to be potentially associated with differences in non-coding repetitive DNA sequences, for example, in *Z. mays* L. [34], *C. quinoa* [32], and *B. rapa* [66]. Therefore, in our study, we proposed that the observed variation in the saffron (*C. sativus*) genome among different genotypes may be due to the contribution of repetitive sequences.

Although saffron is reproduced exclusively through clonal propagation, genetic diversity has been detected among saffron accessions using ITS2 and *trnH–psbA* DNA barcodes [14]. In addition, analyses based on SSR and SRAP markers have revealed genetic variation both among and within populations [18,69]. In this context, both molecular marker-derived information and polymorphisms in nuclear DNA content can serve as complementary indicators of genetic diversity and guide future studies aimed at germplasm conservation and resource preservation.

## 5. Conclusions

Assessing genetic diversity in *C. sativus* is essential for biodiversity conservation and crop improvement. Our study indicated statistically significant variations in nuclear genome size among *C. sativus* populations from Safranbolu, which may reflect genomic diversity. These differences may be associated with varying retrotransposon content and suggest minor genetic differentiation among some individuals. Such variation could be valuable for future cytogenetic and molecular analyses, and beneficial for breeding efforts aimed at improving this important crop. Future studies incorporating a larger number of samples and the use of highly polymorphic molecular markers such as SSRs (simple sequence repeats) and SNPs (single nucleotide polymorphisms) would be valuable for gaining a more detailed understanding of genetic relationships among *Crocus sativus* genotypes. These approaches would complement genome size data and enhance insights into the genetic diversity and differentiation of saffron populations. These approaches would complement genome size data and enhance insights into the genetic diversity and differentiation of saffron populations.

## Figures and Tables

**Table 1:** Nuclear DNA content of the analysed samples (pg/2C).

Population Number		Mean Nuclear DNA Content	Standard Deviation	Confidence Interval	
				Min	Max
9	Davutobası	10.85	0.07	10.77	10.89
11	Davutobası	10.9	0.09	10.82	11
10	Davutobası	10.56	0.12	10.47	10.69
2	Yukarıçiftlik	10.59	0.04	10.55	10.63
15	Davutobası	10.84	0.04	10.81	10.88
8	Davutobası	10.89	0.17	10.7	11.03
4	Yukarıçiftlik	10.72	0.07	10.68	10.8
14	Davutobası	10.64	0.06	10.57	10.67
6	Davutobası	10.45	0.08	10.36	10.5
12	Davutobası	10.65	0.02	10.64	10.67
1	Yukarıçiftlik	10.71	0.03	10.68	10.74
3	Yukarıçiftlik	10.83	0.07	10.75	10.87
7	Davutobası	10.87	0.15	10.7	10.97
13	Davutobası	10.79	0.12	10.69	10.93
5	Yukarıçiftlik	10.58	0.12	10.46	10.69

## Data Availability

The original contributions presented in this study are included in the article. Further inquiries can be directed to the corresponding author.
